# The pancreas-to-muscle signal intensity ratio on T_1_-weighted MRI as a predictive biomarker for postoperative pancreatic fistula after distal pancreatectomy: a single-center retrospective study

**DOI:** 10.1186/s12957-022-02718-8

**Published:** 2022-08-05

**Authors:** Masahiro Fukada, Katsutoshi Murase, Toshiya Higashi, Seito Fujibayashi, Masashi Kuno, Itaru Yasufuku, Yuta Sato, Shigeru Kiyama, Yoshihiro Tanaka, Naoki Okumura, Nobuhisa Matsuhashi, Takao Takahashi

**Affiliations:** Department of Gastroenterological Surgery, Gifu University Hospital, 1-1 Yanagido, Gifu City, Gifu 501-1194 Japan

**Keywords:** Signal intensity ratio on T_1_-weighted MRI, Postoperative pancreatic fistula, Distal pancreatectomy

## Abstract

**Background:**

Postoperative pancreatic fistula (POPF) is one of the serious complications of pancreatic surgery. When POPF occurs and becomes severe, it causes secondary complications and a longer treatment period. We previously reported a correlation between pancreatic fibrosis and magnetic resonance imaging (MRI) findings, and MRI may have the potential to predict POPF. This study aimed to assess the predictive ability of the pancreas-to-muscle signal intensity ratio on T_1_-weighted MRI (SIR on T_1_-w MRI) for POPF after distal pancreatectomy (DP).

**Methods:**

This single-institution retrospective study comprised 117 patients who underwent DP. It was conducted between 2010 and 2021 at the Gifu University Hospital. We statistically analyzed pre-, intra-, and postoperative factors to assess the correlation with POPF.

**Results:**

According to the definition and grading of the International Study Group of Pancreatic Fistula (ISGPF), 29 (24.8%) of the 117 patients had POPF grades B and C. In the univariate analysis, POPF was significantly associated with the pancreas-to-muscle SIR on T_1_-w MRI, the drainage fluid amylase concentration (D-Amy) levels on postoperative day (POD) 1 and 3, white blood cell count on POD 1 and 3, C-reactive protein level on POD 3, and heart rate on POD 3. In multivariate analysis, only the pancreas-to-muscle SIR on T_1_-w MRI (>1.37; odds ratio [OR] 23.25; 95% confidence interval [CI] 3.93–454.03; *p* < 0.01) and D-Amy level on POD 3 (>737 U/l; OR 3.91; 95% CI 1.02–16.36; *p* = 0.046) were identified as independent predictive factors.

**Conclusions:**

The pancreas-to-muscle SIR on T_1_-w MRI and postoperative D-Amy levels were able to predict the development of POPF after DP. The pancreas-to-muscle SIR on T_1_-w MRI may be a potential objective biomarker reflecting pancreatic status.

## Background

Postoperative pancreatic fistula (POPF) is one of the most serious complications of pancreatic surgery for pancreatic disease. POPF causes secondary complications, such as abdominal abscess, delayed gastric emptying, and postoperative bleeding, and may lead to a prolonged hospital stay duration and surgery-related death [1–3]. Although surgical procedures have been standardized and surgical devices developed in pancreatic surgery, the incidence of POPF has been reported to still range from 3 to 50%, even at high-volume centers [4–7]. Furthermore, POPF still occurs at, as high as, 24 to 39% even after distal pancreatectomy (DP) without pancreaticoenteral anastomosis [8–13]. Therefore, POPF is considered to be caused by surgery-related factors and pancreas-related factors.

Pancreatic parenchyma becomes hardened because of fibrosis, and the hardness of the pancreatic parenchyma is known to be associated with POPF [14, 15]. We previously reported a significant correlation between the pathological classification of the pancreatic fibrosis grade and the development of POPF [16, 17]. We also reported using the pancreas-to-muscle signal intensity ratio on T1-weighted MRI (SIR on T1-w MRI) to evaluate pancreatic fibrosis and predict POPF [16, 17].

This study aimed to assess the potential of the pancreas-to-muscle SIR on T_1_-w MRI as a predictive factor for POPF after DP.

## Methods

### Patients

**Fig. 1:**
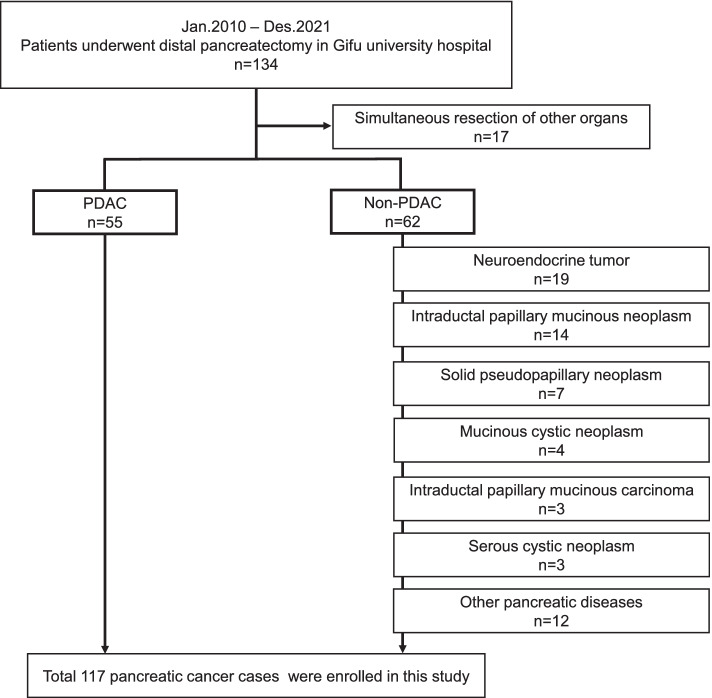
Exclusion criteria

In this single-center retrospective study, we included 134 consecutive patients who underwent DP for pancreatic disease at Gifu University Hospital between January 2010 and December 2021. All procedures were conducted by expert surgeons who had qualified through the board certification system of the Japanese Society of Hepato-Biliary-Pancreatic Surgery (JSHBPS). We excluded 17 patients in total (simultaneous resection of other organs), so 117 patients were included in this study (Fig. 1). We conducted our study in accordance with the World Medical Association Declaration of Helsinki, and the Ethics Committee of Gifu University approved the study (approval number: 2021-026).

**Fig. 2:**
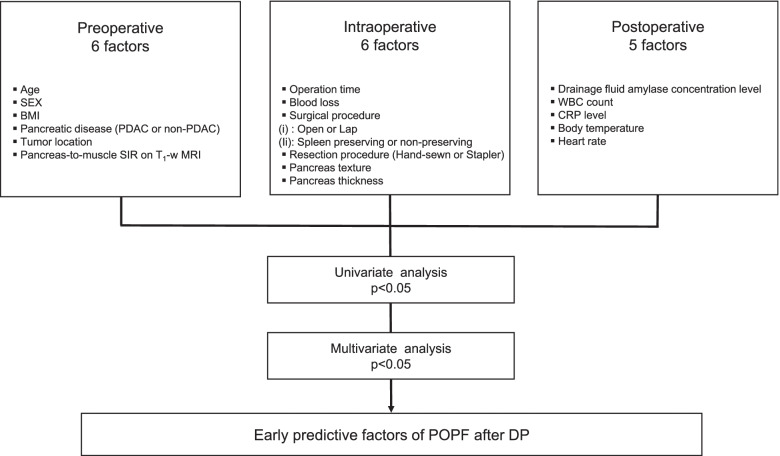
Analysis flow chart for identifying predictive factors for postoperative pancreatic fistula (POPF) after distal pancreatectomy (DP)

Patient characteristics were classified into three categories: pre-, intra-, and postoperative factors (Fig. 2). The six preoperative factors were age, sex, body mass index (BMI), pancreatic disease (pancreatic ductal adenocarcinoma (PDAC) or non-PDAC), tumor location, and the pancreas-to-muscle SIR on T_1_-w MRI. The six intraoperative factors included operative time, blood loss, surgical procedure ((i) open or laparoscopic surgery, (ii) spleen preserving or non-preserving), pancreatic resection procedure (hand-sewn or stapler), pancreas texture (soft or hard), and pancreas thickness measured intraoperatively on resection site. Finally, the five postoperative factors included the amylase concentration levels of drainage fluid (D-Amy), the white blood cell (WBC) count, C-reactive protein (CRP) level, body temperature, and heart rate on postoperative day (POD) 1 and 3. Body temperature was the maximum value and heart rate was the average value on the measurement day.

### Perioperative management

In cases of DP for PDAC, regional lymph node dissection with splenectomy following the classification of pancreatic carcinoma of the Japan Pancreas Society [18] and pancreatic resection of the portal vein were performed. In the case of DP for non-PDAC, systematic lymph node dissection was omitted, and pancreatic resection was performed with sufficient margin from the tumor. Pancreatic resection was performed with a hand-sewn closure or using a linear stapler.

In the hand-sewn closure group, the pancreas was resected after identifying the main pancreatic duct, and the main pancreatic duct was ligated with a 3-0 silk suture. The stump of the remnant pancreas was closed with a vertical mattress suture using 5-0 polypropylene. For the group that underwent pancreatic resection using a linear stapler, the pancreas was resected with a purple or black cartridge using Endo GIA™ Tri-Staple or Signia™ stapling system (Medtronic plc., Dublin, Ireland). The closed jaw was clamped carefully and slowly, taking 5 min, at a fixed speed. The firing was performed at 1 cm per minute by firmly fixing the stapler. After firing, the jaws of the stapler were held shut for 1 min. One 19Fr. Blake silicon drain (Johnson and Johnson Inc. New Brunswick, NJ, USA) was then placed near the stump of the remnant pancreas. The drain was removed on POD 4–5, when the drainage fluid was clear, the postoperative course was stable, and the patient was without abdominal pain, fever, or other symptoms. The D-Amy levels were measured on POD 1, 3, and 5. All patients received prophylactic antibiotics (cefmetazole) only intraoperatively or 2 days postoperatively.

### The pancreas-to-muscle signal intensity ratio on T_1_-weighted MRI

**Fig. 3:**
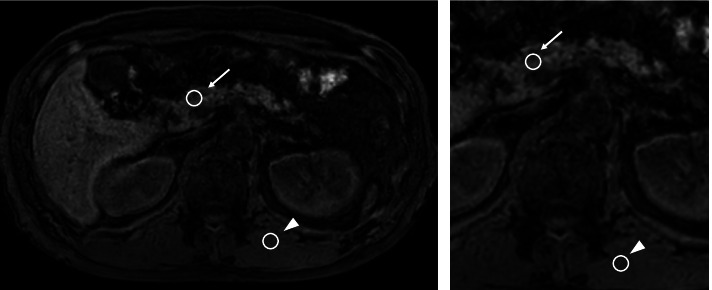
The pancreas-to-muscle signal intensity ratio on fat-suppressed axial T_1_-weighted MRI was calculated by [signal intensity of the pancreatic parenchyma] (arrow)/[signal intensity of the paraspinal muscle] (arrowhead)

Previously, we studied the potential value of preoperative MRI in evaluating pancreatic properties [16, 17] and reported that the pancreas-to-muscle SIR on T_1_-w MRI significantly correlated with pancreatic fibrosis and that it may be a potential biomarker for predicting POPF for pancreatic surgery. The pancreatic parenchyma’s signal intensity (SI) on the portal vein and the paraspinal muscle was measured using fat-suppressed axial T_1_-weighted imaging (Fig. 3). The pancreas-to-muscle SIR on T_1_-w MRI was calculated using the following equation: [SI of the pancreatic parenchyma]/[SI of the paraspinal muscle].

### Definition of POPF

In this study, we included only clinically symptomatic POPF. Therefore, only grades B and C pancreatic fistulas were defined as POPF (grade B, symptomatic fistula requiring therapeutic intervention such as antibiotics and percutaneous drainage; grade C, symptomatic fistula associated with a severe general condition of patients, sepsis, and multiorgan failure requiring aggressive treatment in the intensive care unit and surgical intervention), based on the International Study Group of Pancreatic Fistula (ISGPF) definitions [19]. Diagnosis day of POPF was defined as when intra-abdominal fluid collection with positive cultures was identified by ultrasonography (US) or computed tomography (CT).

### Statistical analysis

Continuous variables were expressed as median (range) values, and categorical variables were expressed as frequencies (percentages). For comparisons of variables between the POPF and non-POPF groups, Fisher’s exact test was used for categorical variables. A Mann-Whitney *U* test was used for continuous variables. The predictive ability for POPF after DP was assessed by calculating the area under the receiver operating characteristic (ROC) curve. Youden’s index was used to determine the optimal cutoff value to calculate specificities and sensitivities in the ROC curve analysis. The variables identified as potentially significant by univariate analysis were selected for multivariate analysis with a logistic regression model to identify the independent predictors of POPF after DP. The limit of statistical significance for all analyses was defined as a 2-sided *p*-value of 0.05. All statistical analyses were performed using JMP software (SAS Institute Inc., Cary, NC, USA).

## Results

### Comparison of clinical outcomes between patients with and without POPF

In total, 117 patients underwent DP for pancreatic disease. Symptomatic POPF occurred in 29 (24.8 %) patients. Patients’ clinical outcomes after DP are summarized in Table 1. The median time at which POPF was confirmed was POD 7 (3–25 days). In the patients with POPF, the median time until hospital discharge was 35 days postoperatively (range, 12–121 days), and two patients had died within 30 postoperative days. A comparison between patients with and without POPF indicated significant differences in hospital stay duration (*p* < 0.01).

**Table 1:** Comparison of clinical outcomes between patients with and without POPF after distal pancreatectomy

	Patients with POPF (*n* = 29)		Patients without POPF (*n* = 88)	*p*-value
Diagnosis days of POPF (day)	7 (3–25)		-	-
Grade of POPF^a^				-
Grade B	27 (93.1)		-	
Grade C	2 (6.9)		-	
Treatment for POPF	・Drain replacement and irrigation	19 (65.5)	-	-
	・Endoscopic transgastric drainage	7 (24.1)		
	・Antibiotics and octreotide	3 (10.4)		
Postoperative death within 30 days	2 (6.9)		0 (0.0)	0.06
Hospital days (days)	35 (12–121)		13 (7–23)	<0.01^✝^

Data are expressed as median (range) or number of patients (percentage)

*POPF* Postoperative pancreatic fistula

^a^International Study Group (ISGPS) definition and grading of POPF as follows: grade B, symptomatic fistula requiring therapeutic intervention such as antibiotics and percutaneous drainage; grade C, symptomatic fistula associated with a severe general condition of patients, sepsis, and multiorgan failure requiring aggressive treatment in the intensive care unit and surgical intervention

^✝^*p* < 0.05

### Comparison of pre-, intra-, and postoperative status between patients with and without POPF

Table 2 summarizes the 17 factors (classified into three categories) compared between patients with and without POPF.

**Table 2:** Comparison of pre-, intra-, and postoperative status between patients with and without POPF after distal pancreatectomy

		Patients with POPF (*n*=29)	Patients without POPF (*n*=88)	*p*-value
Preoperative	Age (years)	67 (40–82)	67 (11–84)	0.87
	Sex			
	Male	18 (62.1)	50 (56.8)	0.67
	Female	11 (37.9)	38 (43.2)	
	BMI (kg/m^2^)	23.6 (17.6–32.2)	22.4 (16.2–32.2)	0.37
	Pancreatic ductal adenocarcinoma	12 (41.4)	43 (48.9)	0.53
	Location			
	Body	14 (48.3)	54 (61.4)	0.28
	Tail	15 (51.7)	34 (38.6)	
	Pancreas-to-muscle SIR on T_1_-w MRI	1.64 (1.25–2.68)	1.35 (0.74–2.16)	<0.01^✝^
Intraoperative	Operative time (min)	284 (174–537)	264 (143–564)	0.13
	Blood loss (ml)	190 (10–1910)	260 (0–1840)	0.84
	Surgical procedure			
	Open	23 (79.3)	60 (68.2)	0.35
	Laparoscopic	6 (20.7)	28 (31.8)	
	Spleen preserving	3 (10.3)	15 (17.1)	0.56
	Non-spleen preserving	26 (89.7)	73 (82.9)	
	Resection procedure			
	Hand-sewn	11 (37.9)	36 (40.9)	0.83
	Stapler	18 (62.1)	52 (59.1)	
	Pancreas texture			
	Soft	21 (72.4)	54 (61.4)	0.37
	Hard	8 (27.6)	34 (38.6)	
	Pancreas thickness (mm)	11 (8–17)	12 (3–24)	0.46
Postoperative	D-Amy levels (U/l)			
	POD 1	7652 (108–34076)	1899 (42–61075)	0.02^✝^
	POD 3	1290 (42–16515)	403 (35–43873)	<0.01^✝^
	WBC (×10^3^/μl)			
	POD 1	12.6 (7.5–26.5)	11.1 (5.3–18.2)	<0.01^✝^
	POD 3	13.3 (5.9–26.9)	11.3 (3.9–23.9)	0.048^✝^
	CRP (mg/dl)			
	POD 1	9.3 (1.8–15.4)	8.3 (0.2–17.5)	0.16
	POD 3	20.1 (8.0–33.5)	14.5 (0.3–26.5)	<0.01^✝^
	Body temperature (°C)			
	POD 1	38.0 (37.1–39.4)	38.0 (36.9–39.3)	0.55
	POD 3	37.4 (36.1–38.9)	37.4 (36.2–39.4)	0.30
	Heart rate (bpm)			
	POD 1	98 (81–142)	92 (61–122)	0.07
	POD 3	88 (72–111)	82 (56–119)	<0.01^✝^

Data are expressed as median (range) or number of patients

*POPF* Postoperative pancreatic fistula, *BMI* Body mass index, *pancreas-to-muscle SIR on T*_*1*_*-w MRI* the pancreas-to-muscle signal intensity ratio on unenhanced T_1_-weigthed magnetic resonance imaging, *D-Amy* drainage fluid amylase concentration, *POD* Postoperative day, *WBC* White blood cell, *CRP* C-reactive protein

^✝^*p*< 0.05

Among preoperative factors, the pancreas-to-muscle SIR on T_1_-w MRI was significantly higher in the POPF group than in the non-POPF group (*p* < 0.01). Among intraoperative factors, there was no significant difference between the two groups. Among postoperative factors, the D-Amy level on POD 1 and 3 (*p* = 0.02 and *p* < 0.01, respectively), WBC on POD 1 and 3 (*p* < 0.01 and *p* = 0.048, respectively), CRP level on POD 3 (*p* < 0.01), and heart rate on POD 3 (*p* < 0.01) were significantly higher in the POPF group than in the non-POPF group.

### Cutoff values of the pancreas-to-muscle SIR on T_1_-w MRI and D-Amy levels for predicting POPF

**Fig. 4:**
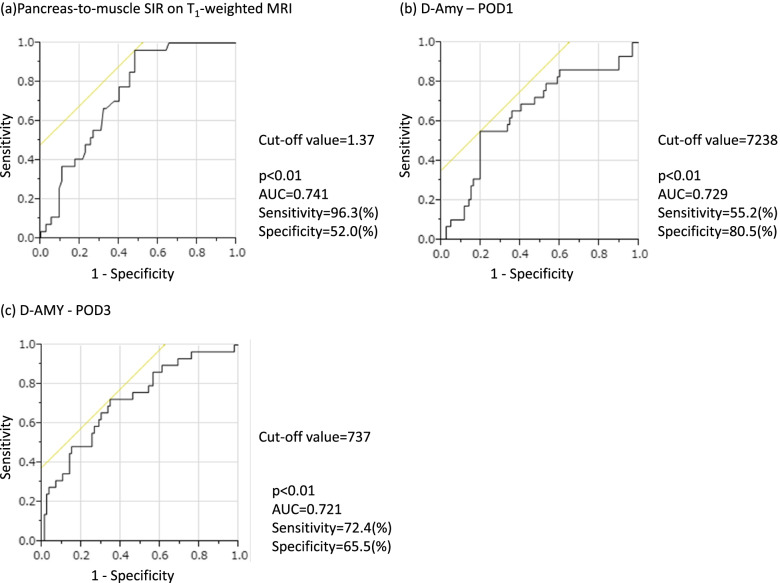
Receiver operating characteristic (ROC) curve analysis of pancreas-to-muscle SIR on T_1_-weighted MRI and D-Amy level on POD 1 and 3 for discriminating to POPF

The ROC curves for generating the cutoff values of the pancreas-to-muscle SIR on T_1_-w MRI and D-Amy level on POD 1 and 3 are shown in Fig. 4. The cutoff value of the pancreas-to-muscle SIR on T_1_-w MRI was +1.37, with an area under the curve (AUC) of 0.741, a sensitivity of 96.3%, and specificity of 52.0% (Fig. 4a). The cutoff value of the D-Amy level on POD 1 was 7238 U/l, with an AUC of 0.729, a sensitivity of 55.2%, and a specificity of 80.5% (Fig. 4b). The cutoff value of the D-Amy level on POD 3 was 737 U/l, with an AUC of 0.721, a sensitivity of 72.4%, and a specificity of 65.5% (Fig. 4c).

### Uni- and multivariate analysis of prediction for POPF after DP

In a univariate logistic regression analysis, POPF after DP was significantly associated with the pancreatic-to-muscle SIR on T_1_-w MRI (*p* < 0.01), D-Amy level on POD 1 and 3 (both *p* < 0.01), WBC on POD 1 and 3 (*p* < 0.01 and *p* = 0.04, respectively), CRP level on POD 3 (*p* < 0.01), and heart rate on POD 3 (*p* = 0.02).

A multivariate logistic regression analysis revealed that the pancreas-to-muscle SIR on T_1_-w MRI (>1.37; odds ratio [OR] 23.25; 95% confidence interval [CI] 3.93–454.03; *p* < 0.01) and D-Amy level on POD 3 (>737 U/l; OR 3.91; 95% CI 1.02–16.36; *p* = 0.046), were independent predictive factors of POPF after DP (Table 3).

**Table 3:** Uni- and multivariate predictive factors of POPF after DP

		*n*	Univariate			Multivariate		
			OR	95% CI	*p*-value	OR	95% CI	*p*-value
Preoperative	Age (years)							
	>70	45	0.79	0.32–1.89	0.61			
	<70	72	1					
	Sex							
	Male	68	1.24	0.53–3.01	0.62			
	Female	49	1					
	BMI (kg/m^2^)							
	>24	39	2.07	0.85–5.00	0.10			
	<24	77	1					
	Pancreatic ductal adenocarcinoma							
	Yes	55	0.74	0.31–1.72	0.48			
	No	62	1					
	Location							
	Body	68	0.59	0.25–1.37	0.22			
	Tail	49	1					
	Pancreas-to-muscle SIR on T_1_-w MRI							
	>1.37	61	29.7	5.83–543.73	<0.01^✝^	23.25	3.93–454.03	<0.01^✝^
	<1.37	41	1			1		
Intraoperative	Operative time (min)							
	>300	38	1.36	0.55–3.24	0.50			
	<300	78	1					
	Blood loss (ml)							
	>400	39	0.86	0.33–2.07	0.73			
	<400	77	1					
	Surgical procedure							
	Open	83	1.79	0.69–5.28	0.24			
	Laparoscopic	34	1					
	Spleen preserving	18	0.56	0.12–1.87	0.37			
	Non-spleen preserving	99	1					
	Resection procedure							
	Hand-sewn	47	0.88	0.36–2.07	0.78			
	Stapler	70	1					
	Pancreas texture							
	Soft	75	1.65	0.68–4.35	0.28			
	Hard	42	1					
	Pancreas thickness (mm)							
	>12	56	0.71	0.30–1.64	0.42			
	<12	61	1					
Postoperative	D-Amy levels (U/l)—POD 1							
	>7238	33	5.07	2.07–12.78	<0.01^✝^	1.21	0.31–4.75	0.78
	<7238	83	1			1		
	D-Amy levels (U/l)—POD 3							
	>737	51	4.99	2.04–13.25	<0.01^✝^	3.91	1.02–16.36	0.046^✝^
	<737	65	1			1		
	WBC (×10^3^/μl)—POD 1							
	>1.20	48	3.16	1.34-7.75	<0.01^✝^	2.44	0.69–9.12	0.17
	<1.20	69	1			1		
	WBC (×10^3^/μl)—POD 3							
	>1.40	34	2.59	1.07–6.29	0.04^✝^	1.03	0.27–3.73	0.96
	<1.40	83	1			1		
	CRP (mg/dl)—POD 1							
	>10	40	1.51	0.63–3.58	0.35			
	<10	77	1					
	CRP (mg/dl)—POD 3							
	>20	34	3.89	1.61–9.60	<0.01^✝^	2.75	0.75–10.70	0.13
	<20	83	1			1		
	Body temperature (°C)—POD 1							
	>38.0	71	0.74	0.32–1.75	0.49			
	<38.0	46	1					
	Body temperature (°C)—POD 3							
	>38.0	14	0.47	0.07–1.87	0.31			
	<38.0	103	1					
	Heart rate (bpm)—POD 1							
	>100	38	2.05	0.85–4.88	0.11			
	<100	79	1					
	Heart rate (bpm)—POD 3							
	>100	13	4.35	1.32–14.81	0.02^✝^	3.84	0.77–21.40	0.10
	<100	104	1			1		

*POPF* Postoperative pancreatic fistula, *OR* Odds ratio, *95% CI* 95% Confidence interval, *BMI* Body mass index, *pancreas-to-muscle SIR on T*_*1*_*-w MRI* the pancreas-to-muscle signal intensity ratio on unenhanced T_1_-weigthed magnetic resonance imaging, *D-Amy* drainage fluid amylase concentration, *POD* Postoperative day, *WBC* White blood cell, *CRP* C-reactive protein

^✝^*p* < 0.05

## Discussion

A high incidence of POPF is still reported in pancreatic surgery despite ongoing attempts to reduce its frequency with the development of surgical techniques and devices [4–13]. The clinical nuisance of POPF is that delayed therapeutic intervention can lead to secondary complications [1–3]. This can lead to severe disease and prolonged treatment. In this study, patients with POPF showed an increase in hospital stay duration and mortality. Furthermore, we have previously studied that in PDAC cases, the onset of POPF leads to a delay in initiating postoperative adjuvant chemotherapy [20]. Thus, POPF may affect not only short-term, but also long-term prognosis. Therefore, early and accurate diagnosis of POPF and the promptest possible interventions possible are required. However, this study’s median time for POPF diagnosis was 7 days (range, 3–25), making early diagnosis difficult with only routine postoperative examination. We identified two predictive factors for POPF: (i) the pancreas-to-muscle SIR on T_1_-w MRI > 1.37 and (ii) the D-Amy level on POD 3 > 737 U/l.

D-Amy levels are among the most established predictive factors for POPF. Therefore, the definition of POPF according to the ISGPF offers the definitive diagnosis according to the D-Amy level on POD 3. In this study, D-Amy levels were also significantly correlated with POPF. There is no doubt that amylase in the drainage fluid is useful in predicting POPF, as has been reported many times [21–42]. However, the following remain somewhat unclear: (1) the optimal timing of measurement, (2) the optimal cutoff value, (3) the optimal drain placement site, and (4) whether drainage fluid concentration or the total amount of amylase is more reliable. In addition, postoperative drain obstruction due to fibrin or clots, and drain misalignment often occurs, interfering with accurate D-Amy level measurements.

The nature of the pancreas itself is thought to play a profound role in the development of POPF. In particular, the texture of pancreatic parenchyma (soft pancreas) has been an important risk factor for POPF. However, the problem is that the pancreatic texture is very subjective and cannot be quantified. To solve this problem, we previously investigated the correlation between preoperative pancreatic MRI features and the histopathological pancreatic fibrosis grade of surgical specimens (fibrosis was graded as follows: F0 = normal pancreatic parenchyma, no fibrotic changes; F1 = mild fibrosis with thickening of periductal fibrosis tissue; F2 = moderate fibrosis with marked sclerosis of interlobular septa and no evidence of architectural changes; and F3 = severe fibrosis with detection of architectural destruction) [16, 17]. We found that the pancreas-to-muscle SIR on T_1_-w MRI significantly correlated with the pancreatic fibrosis grade. This is because normal pancreatic parenchyma exhibits hyperintensity on T_1_-w MRI, as pancreatic juice is rich in glycoproteins, and the endoplasmic reticulum within the pancreatic cells contributes to the T_1_ shortening effect. However, the signal intensity gradually decreases with the progression of pancreatic atrophy, fibrosis, interstitial edema, or fat deposition [43, 44]. In our previous study, the mean pancreas-to-muscle SIR on T_1_-w MRI values for F0 and F1, which correspond to the soft pancreas, were 1.51 and 1.48, respectively. Furthermore, the pancreas-to-muscle SIR on T_1_-w MRI in patients with POPF was significantly higher than that in patients without POPF. Based on these findings, we hypothesized that the pancreas-to-muscle SIR on T_1_-w MRI might be a potential biomarker for predicting POPF and calculated the cutoff value of 1.41 [16]. Yoon et al. conducted a similar study and reported the mean pancreas-to-muscle SIR on T1-w MRI values for F0 and F1 and the cutoff value for predicting POPF was 1.51, 1.48, and 1.40, respectively [45]. Interestingly, the calculated cutoff value for predicting POPF (1.37) in this study is very close to previous studies.

This study had some limitations. First, it was retrospective in design, was undertaken at a single institution, and involved a small number of study patients. The relatively small sample size may have caused a selection bias and multiplicity issues in statistical analysis. This limitation should be considered when evaluating our study results. A prospective, multi-centered study is needed involving a larger number of patients in the future. Second, there were the technical variations in the surgical procedure of DP, such as open or laparoscopic, spleen preserving or non-preserving, hand-sewn or stapler, and lymph node dissection or not. This study found no significant correlation between surgical-related factors and POPF. It is necessary to unify surgical techniques in order to calculate more appropriate cutoff values of the pancreas-to-muscle SIR on T1-weighted MRI and D-Amy levels.

## Conclusions

We found that the pancreas-to-muscle SIR on T_1_-weighted MRI and D-Amy levels may have predictive value for POPF. The pancreas-to-muscle SIR is an objective and quantitative biomarker reflecting pancreatic characteristics. Postoperative management based on the pancreas-to-muscle SIR on T_1_-weighted MRI may contribute to a shortened hospital stay.

## Data Availability

The datasets used during this study are available from the corresponding author upon reasonable request.

## Abbreviations

BMI: Body mass index
CI: Confidence interval
CT: Computed tomography
D-Amy: Drainage fluid amylase
DP: Distal pancreatectomy
MRI: Magnetic resonance imaging
OR: Odds ratio
ISGPF: International Study Group of Pancreatic Fistula
PD: Pancreaticoduodenectomy
PDAC: Pancreatic ductal adenocarcinoma
POD: Postoperative day
POPF: Postoperative pancreatic fistula
ROC: Receiver operating characteristic
SIR on T_1_-w MRI: Signal intensity ratio on T_1_-weighted MRI
US: Ultrasonography
